# Enhanced VEGF/VEGF-R and RUNX2 Expression in Human Periodontal Ligament Stem Cells Cultured on Sandblasted/Etched Titanium Disk

**DOI:** 10.3389/fcell.2020.00315

**Published:** 2020-05-14

**Authors:** Guya Diletta Marconi, Francesca Diomede, Jacopo Pizzicannella, Luigia Fonticoli, Ilaria Merciaro, Sante D. Pierdomenico, Emanuela Mazzon, Adriano Piattelli, Oriana Trubiani

**Affiliations:** ^1^Department of Medical, Oral and Biotechnological Sciences, “G. d’Annunzio” University of Chieti-Pescara, Chieti, Italy; ^2^ASL 02 Lanciano-Vasto-Chieti, SS. Annunziata Hospital, Chieti, Italy; ^3^IRCCS Centro Neurolesi “Bonino-Pulejo,” Messina, Italy

**Keywords:** titanium disks, angiogenesis, osseointegration, mesenchymal stem cell, cytocompatibility, human periodontal ligament stem cells

## Abstract

Bone formation, in skeletal development or in osseointegration processes, is the result of interaction between angiogenesis and osteogenesis. To establish osseointegration, cells must attach to the implant in a direct way without any deposition of soft tissue. Structural design and surface topography of dental implants enhance the cell attachment and can affect the biological response. The aim of the study was to evaluate the cytocompatibility, osteogenic and angiogenic markers involved in bone differentiation of human periodontal ligament stem cells (hPDLSCs) on different titanium disks surfaces. The hPDLSCs were cultured on pure titanium surfaces modified with two different procedures, sandblasted (Control—CTRL) and sandblasted/etched (Test—TEST) as experimental titanium surfaces. After 1 and 8 weeks of culture VEGF, VEGF-R, and RUNX2 expression was evaluated under confocal laser scanning microscopy. To confirm the obtained data, RT-PCR and WB analyses were performed in order to evaluate the best implant surface performance. TEST surfaces compared to CTRL titanium surfaces enhanced cell adhesion and increased VEGF and RUNX2 expression. Moreover, titanium TEST surfaces showed a different topographic morphology that promoted cell adhesion, proliferation, and osteogenic/angiogenic commitment. To conclude, TEST surfaces performed more efficiently than CTRL surfaces; furthermore, TEST surface results showed them to be more biocompatible, better tolerated, and appropriate for allowing hPDLSC growth and proliferation. This fact could also lead to more rapid bone–titanium integration.

## Introduction

In the last few years, mesenchymal stem cells (MSCs) have been performing a primary function in regenerative medicine and tissue engineering. They offer the opportunity to restore, recover, and renovate cells, tissues, and organs (Di Nisio et al., 2015). In dentistry, human periodontal ligament stem cells (hPDLSCs) have been the mostly utilized MSCs population. They have been collected from the periodontal ligament, a specialized connective tissue that linked alveolar bone socket with the tooth root surface, distinguished by various cell populations as osteoblasts, fibroblasts, epithelial cells, endothelial cells, and stem cells (De Colli et al., 2015; Safi et al., 2019). The hPDLSCs show multipotent and proliferative properties (Winning et al., 2017). In fact, hPDLSCs improved the interface formation among dental devices utilized for implants and bone in an event defined osseointegration (Kado et al., 2019). Devices used in dentistry are composed by materials with chemical and physical properties able to differentiate stem cells into osteoprogenitor cells (Kim et al., 2014). Titanium is one of the materials mostly used in this field. It has a good biocompatibility, is relatively inert, and is defined by surface features such as topography, roughness, and hydrophilicity that support cell growth and differentiation, influence the protein adsorption of the device implant and its immobilization (Gittens et al., 2014; Liu et al., 2017; Selders et al., 2017; Yadav et al., 2017). One of the primary aims of research in dentistry has been to improve the bone–implant interface and the titanium bioactivity by modifying its surface structure. The formation of a direct contact between the implant and mineralized bone is the result of angiogenic and osteogenic differentiation processes (Hanawa, 2019). There is no bone formation without a prior formation of newly -formed blood vessels. The existence of blood vessels shows a vital role in bone renewal processes and in implant osseointegration (Beltran-Partida et al., 2017; Genova et al., 2019; Raines et al., 2019b). The relationship between the complex molecular mechanisms of blood vessels and bone formation is still relatively unexplored, and a better understanding of these features would be welcomed. Recent studies have reported a close relationship between MSCs and endothelial cell, improving vasculogenesis (Genova et al., 2019; Raines et al., 2019b). It has been hypothesized that changes in the microstructure of implant surface could have a relationship with the secretion by osteoprogenitor cells of pro-angiogenic substances (Raines et al., 2019a). The modification of titanium implant surfaces, by chemical or physical treatment, could represent an effective method, affecting cell adhesion and differentiation (Zahran et al., 2016). In the oral cavity, numerous typologies of MSCs have been described (Rajan et al., 2016; Bianchi et al., 2017; Diomede et al., 2018c). As extensively reported in the literature, MSCs can be easily isolated form craniofacial bones during routine dentistry procedures. Owing to their embryological origin from neural crest, they appear as a suitable cell population to evaluate cell–biomaterial connection in the craniofacial field, involving osteoinductive/osteointegrative events. The biological and immunomodulatory characteristics of MSCs could be affected by chemistry and topography of implant surfaces (Conserva et al., 2019). Until now, six different human dental stem cells have been reported in the literature: human dental pulp stem cells (DPSCs), human exfoliated deciduous teeth stem cells (SHED), hPDLSCs (Sinjari et al., 2019), human apical papilla stem cells (APSCs), human dental follicle stem cells (DFSCs), and human gingival GMSCs (Romeo et al., 2018; Trubiani et al., 2019b). In particular, MSCs taken from the human periodontal ligament can be easily isolated and manipulated to use as an *in vitro* model to assess cell cytocompatibility of different materials (Diomede et al., 2016; Pizzicannella et al., 2018b). Moreover, the complex restoration of the periodontal ligament has shown unexpected clinical results and preserves an attractive challenge in dentistry (Trubiani et al., 2012b; Pizzicannella et al., 2018a). The goal of the current work was to evaluate the presence of angiogenic and osteogenic markers as RUNX2 and VEGF, and their receptors, on different titanium disks surfaces.

## Materials and Methods

### Ethics Statement

The protocol and informed consent from human periodontal ligament biopsies were accepted by the Medical Ethics Committee at the Medical School, “G. d’Annunzio” University, Chieti, Italy (no. 266/17.04.14). The formal consent form was subscribed by all patients prior to sample collection. The Department of Medical, Oral and Biotechnological Sciences and the Laboratory of Stem Cells and Regenerative Medicine are certified in accordance with the quality standard ISO 9001:2015 (certificate no. 32031/15/S).

### Cell Culture

Five human periodontal ligament biopsies were scraped from human premolar teeth of patients in general good health conditions. The tissue was obtained by scaling the roots using Gracey’s curettes (Trubiani et al., 2012a; Diomede et al., 2018b). The samples were washed five times with PBS (LiStarFish) and cultured using TheraPEAK^TM^ MSCGM^TM^ CD BulletKit serum free, chemically defined (MSCGM-CD) medium for the growth of human MSCs (Lonza, Basel, Switzerland) (Libro et al., 2016). The medium was changed twice a week, and cells migrating from the explants tissue after reaching about 80% of confluence, were trypsinized (LiStarFish), and after, were subcultured until passage 2nd (P2).

### hPDLSC Characterization

The study of hPDLSC phenotype was performed by flow cytometry, as earlier stated (Diomede et al., 2018a). Shortly, 2.5 × 10^5^ cells were incubated for 30 min with the following antibodies: anti-CD44-FITC, anti-CD105-FITC, and anti-CD29-PE (Ancell Corporation, Bayport, MN, United States); anti-CD14-FITC (Miltenyi Biotec, Bergisch Gladbach, Germany); OCT3/4-PE, SOX2-Alexa488, CD73-PE,CD90-FITC (Becton Dickinson, San Jose, CA, United States) and CD34-PE (Beckman Coulter, Fullerton, CA, United States). After incubation with proper secondary antibodies, fixation in 1 mlL of PBS 0.5% paraformaldehyde and washing, cells were detected utilizing a FACStar -plus flow cytometry system and the FlowJo^TM^ software v10.0.7 (Tree Star, Ashland, OR, United States). The hPDLSCs at P2 were analyzed with an inverted light microscopy Leica DMIL (Leica Microsystem, Milan, Italy).

### Dental Implants

In the current work, two different titanium disk surfaces, provided by Implacil De Bortoli (São Paulo, Brazil), have been utilized: Control (CTRL) and Test (TEST). The disks were manufactured of commercially pure titanium (ASTM F67). The surface of CTRL disks was obtained by sandblasting with a mix of titanium oxide power and then cleaning with purified water, enzymatic detergent, acetone, and alcohol, while the surface of TEST disks, after the same sandblasting procedure, was cleaned with purified water, enzymatic detergent, acetone, alcohol, and then a double acid attack with acetylic acid.

### Atomic Force Microscopy (AFM)

The morphologies of two disk surfaces, CTRL and TEST, were assessed by Atomic Force Microscopy (AFM), exploiting a Multimode 8 Bruker AFM microscope (Bruker, Milan, Italy) coupled with a Nanoscope V controller (Bruker AXS, Marne La Vallee, France) and commercial silicon tips (RTESPA 300, resonance frequency of 300 kHz, and nominal elastic constant of 40 N/m) were used in ScanAsyst air mode. The ScanAsyst air mode technique was used for the AFM observations with a scan size of 10 μm. Then the Nanoscope analysis 1.8 software was adopted to analyze images and 3D reconstruction. The roughness average (Ra), that is the arithmetic mean of the absolute values of the height of the surface profile, was assumed for the statistical analysis. Five samples of each group were analyzed, and the mean values (±standard deviation) were considered for statistical analysis (Mastropasqua et al., 2020).

### Scanning Electron Microscopy (SEM) Analysis

CTRL and TEST samples were cultured with hPDLSCs for 21 days and successively were fixed for 4 h at 4°C in 4% Glutaraldehyde in 0.05 M phosphate buffer (pH 7.4), dehydrated in growing ethanol concentrations, and then critical point dried. They were then mounted on aluminum stubs and gold coated in an Emitech K550 (Emitech Ltd., Ashford, United Kingdom) sputter coated before imaging by means of SEM (ZEISS, EVO 50) (Gugliandolo et al., 2018).

### Cell Viability

The cell viability of hPDLSCs seeded with or without CTRL and TEST samples was measured by the quantitative colorimetric MTT [3-(4,5-dimethyl-2-thiazolyl)-2,5-diphenyl-2H-tetrazoliumbromide test] (Promega, Milan, Italy) as earlier described (Cavalcanti et al., 2015). Into a 96-well culture plate with MSCBM medium (Lonza), 2.5 × 10^5^ cells/well were cultured after 24 h of incubation at 37°C, 15 μl/well of MTT was added to culture medium, and cells were incubated for 3 h at 37°C. The supernatants were read at 650-nm wavelength utilizing an ND-1000 NanoDrop Spectrophotometer (NanoDrop Technologies, Rockland, DE, United States). The MTT assay was executed in three independent experiments.

### Alizarin Red S Staining (ARS)

After 1 and 8 weeks of cultures on both CTRL and TEST titanium surfaces, calcium deposition and extracellular matrix mineralization were evaluated by ARS staining assay. This analysis was executed according to the method previously described (Pizzicannella et al., 2019a). Human PDLSCs were washed with PBS, fixed in 10% (*v*/*v*) formaldehyde (Sigma-Aldrich) for 30 min, and washed twice with a lot of dH_2_O prior to the addition of 0.5% Alizarin Red S in H_2_O, pH 4.0, for 1 h at room temperature. Later, cell incubation under gentle shaking was done, and cells were washed with dH_2_O four times for 5 min. For dying quantification, 800 μl of 10% (*v*/*v*) acetic acid was added to each well. Cells were incubated for 30 min with shaking, and then scraped from the plate, moved into a 1.5-ml vial, and vortexed for 30 s. The obtained suspension, overlaid with 500 μl of mineral oil (Sigma-Aldrich), was heated to 85°C for 10 min, then moved to ice for 5 min, meticulously avoiding opening the tubes until fully cooled, and centrifuged at 20,000 × *g* for 15 min. Five hundred microliters of the supernatant was positioned into a new 1.5-ml vial, and 200 μl of 10% (*v*/*v*) ammonium hydroxide was added to neutralize the acid, ensuring a pH between 4.1 and 4.5. One hundred fifty microliters of the supernatant taken from hPDLSCs grown with CTRL and TEST titanium surfaces after 1 and 8 weeks of culture was read in triplicate at 405 nm by a spectrophotometer (Synergy HT). The osteogenic induction was assessed in three independent experiments for each experimental group, and spectrophotometer reads were normalized per cell number.

### Confocal Laser Scanning Microscopy (CLSM) Analysis

Cells grown on CTRL and TEST samples were fixed for 10 min at room temperature (RT) with 4% paraformaldehyde in 0.1 M sodium phosphate buffer (PBS), pH 7.4 (Di Giulio et al., 2015; Mazzatenta et al., 2016). After washing in PBS, cultures were processed for immunofluorescence labeling. Briefly, hPDLSCs grown on titanium disks were permeabilized with 0.5% Triton X-100 in PBS, followed by blocking with 5% skimmed milk in PBS (Giacoppo et al., 2017). Primary monoclonal antibodies to anti human VEGF (Santa Cruz Biotechnology, Santa Cruz, CA, United States), VEGF-R (Santa Cruz Biotechnology), and RUNX2 (Santa Cruz Biotechnology) were utilized, continued by Alexa Fluor 488 green fluorescence-conjugated goat anti-mouse as secondary antibodies (Molecular Probes, Invitrogen, Eugene, OR, United States). Successively, samples were incubated with Alexa Fluor 594 phalloidin red fluorescence conjugate (Molecular Probe), as actin cytoskeleton marker. Nuclei were dyed with TOPRO (Molecular Probe). Samples were put facing down on glass slides and mounted with Prolong antifade (Molecular Probes) (Diomede et al., 2018d). Staining of samples was assessed utilizing a Zeiss (Jena, Germany) LSM510 META confocal system, linked to an inverted Zeiss Axiovert 200 microscope furnished with a Plan Neofluar oil-immersion objective (40×/1.3 NA). Images were analyzed utilizing an argon laser beam with excitation lines at 488 nm. After 1 and 8 weeks of culture, the percentages of VEGF/VEGF-R/RUNX2-positive cells were measured, built on the 15 pictures taken casually. Experiments have been performed in triplicates.

### Gene Expression

VEGF and RUNX2 mRNA expressions were assessed by real-time PCR. For this purpose, total RNA was isolated utilizing the Total RNA Purification kit (NorgenBiotek Corp., Thorold, ON, Canada) in agreement with the manufacturer’s instructions (Zara et al., 2013). The M-MLV Reverse Transcriptase reagents (Applied Biosystems) were utilized to produce cDNA. Real-time PCR was performed with the Mastercycler ep realplex real-time PCR system (Eppendorf, Hamburg, Germany). Expression levels in cells seeded onto CTRL and TEST samples were compared. Commercially available TaqMan Gene Expression Assays (VEGF Hs00900055_m1, VEGF-R Hs00157093_m1, and RUNX2 Hs00231692_m1) and the TaqMan Universal PCR Master Mix (Applied Biosystems, Foster City, CA, United States) were utilized in agreement with the standard protocols (Pizzicannella et al., 2019b). Beta-2 microglobulin (B2M Hs99999907_m1; Applied Biosystems, Foster City, CA, United States) was utilized for template normalization. RT-PCR was analyzed in three independent experiments; duplicate determinations were obtained for each sample.

### Protein Expression

Thirty micrograms of proteins, acquired from all samples, were processed as earlier mentioned (Mammana et al., 2019). Membranes were incubated with primary antibodies rabbit anti-VEGF (1:750; rabbit; Sigma-Aldrich, Milan, Italy), RUNX2 (1:750; rabbit; Sigma-Aldrich), and anti-beta-actin (1:750; mouse; Santa Cruz Biotechnology, Santa Cruz, CA, United States). After five washes in PBS containing 0.1% Tween-20, samples were incubated for 1 h at room temperature with peroxydase-conjugated secondary antibody anti-rabbit and anti-mouse diluted 1:2,000 in 1 × PBS, 3% milk, 0.1% Tween (Mazzatenta et al., 2014). Protein expression was detected using the enhanced chemiluminescence detection system (ECL; Amersham Pharmacia Biotech, Milan, Italy) with photo documenter Alliance 2.7 (Uvitec, Cambridge, United Kingdom). Signals were captured by ECL enhancing and analyzed using a UVIband-1D gel analysis (Uvitec).

### Data and Statistical Analysis

The Statistical Package for Social Science (SPSS, v.21.0, Inc., Chicago, IL, United States) was used for data analysis. Parametrical methods were used after having verified the existence of the required assumptions. In particular, the normality of the distribution and the equality of variances were assessed by the Shapiro–Wilk and Levene’s tests, respectively. Data were expressed as means and standard deviation of the recorded values. The differences among the levels of the factors under investigation were assessed performing three distinct two-way ANOVA tests, one for each experiment. Tukey tests were applied for pairwise comparisons. A value of *p* < 0.05 was considered statistically significant in all tests.

## Results

### Titanium Surface Evaluation at AFM

**FIGURE 1:**
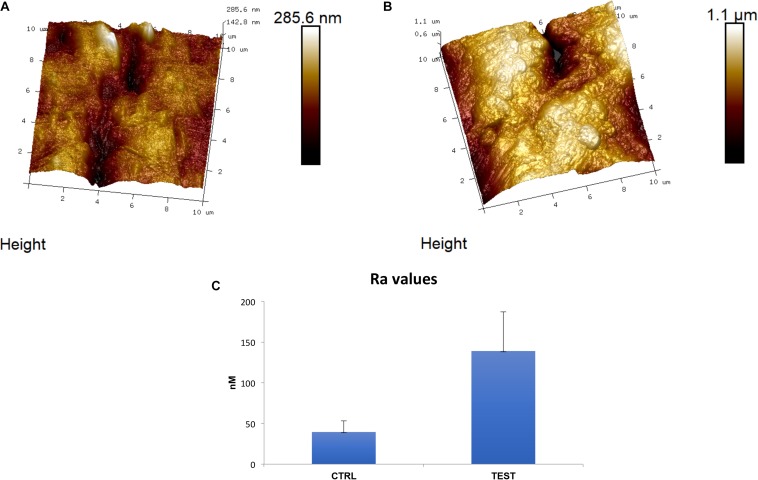
Implant surface evaluation. 3D reconstruction of atomic force microscopy (AFM) images of **(A)** Control (CTRL), **(B)** Test (TEST) surfaces and **(C)** histograms of roughness average (Ra) values.

The two titanium disks surfaces, CTRL and TEST, were analyzed by utilizing AFM. In Figure 1, tridimensional micrographs of the titanium surfaces are studied. From the height panel, the Nanoscope analysis 1.8 software (Bruker, Milan, Italy) is capable of analyzing roughness (Diomede et al., 2018a). The AFM analysis have demonstrated that the TEST group was identified by an average roughness (Ra) of 139 ± 48.1 and 39.5 ± 13.6 nm (Ra) for the CTRL (Figure 1). These results extracted demonstrated that the roughness for the TEST surface is much higher compared to the CTRL titanium surface.

### Human PDLSC Characterization

**FIGURE 2:**
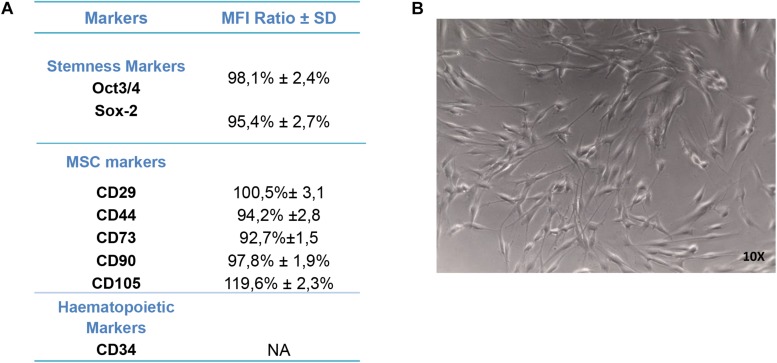
Human periodontal ligament stem cell (hPDLSC) characterization. **(A)** Cytofluorimetric analysis of hPDLSCs. **(B)** hPDLSCs observed at light microscopy (original magnification: 10×).

For the purpose of evaluating the hPDLSC phenotype, flow cytometry analysis was performed on stem cells at the second passage. *Ex vivo* expanded hPDLSCs evidenced positivity for Oct3/4, Sox-2, CD29, CD44, CD73, CD90, and CD105 (Figure 2A). Primary cultures of hPDLSCs at second passage, observed by light microscopy, showed a spindle-shaped morphology (Figure 2B).

### Human PDLSC Adhesion

**FIGURE 3:**
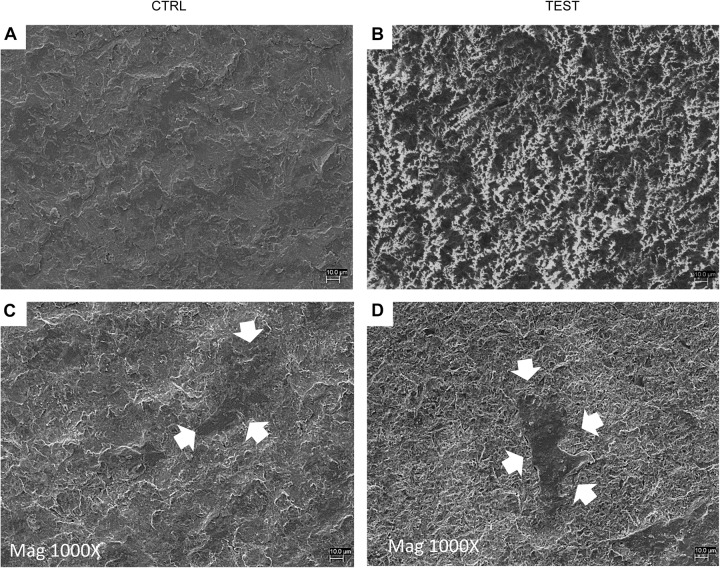
Scanning electron microscopy (SEM) analysis showed the hPDLSCs’ adhesion capacity to the titanium implant surfaces. **(A)** CTRL surface without cells. **(B)** TEST surface without cells. **(C)** Human PDLSCs adhere on CTRL surface. **(D)** Human PDLSCs adhere on TEST surface. Mag: 1,000×. Arrows indicate cells attached on titanium surface.

**FIGURE 4:**
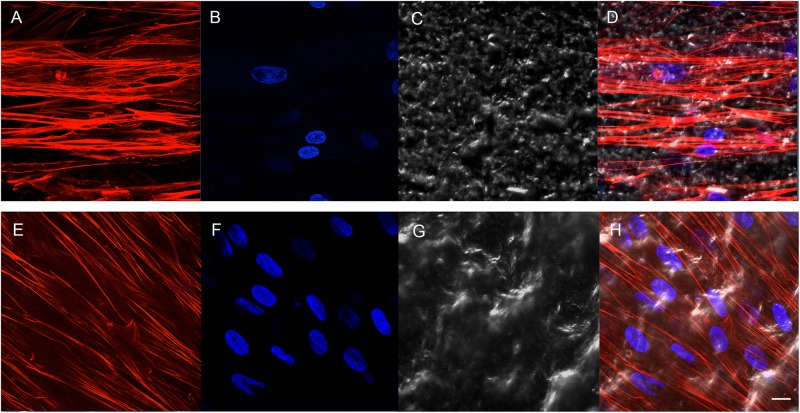
Human PDLSCs seeded on CTRL **(A–D)** and TEST **(E–H)** titanium surfaces for 48 h. **(A,E)** Cytoskeleton actin was dyed in red fluorescence; **(B,F)** nuclei were dyed in blue fluorescence; **(C,G)** TL, transmission light: gray; **(D,H)** merged picture of the abovementioned channels. Scale bar: 10 μm.

Cell adhesion has been evaluated by SEM and CLSM techniques. SEM observations evidenced the surface morphology of two distinct, taken into consideration, surfaces, CTRL and TEST, without cells (Figures 3A,B). Human PDLSCs exhibited the same adhesion capability on CTRL and TEST surfaces not having any morphological variations (Figures 3C,D). At CLSM level, fluorescence figures of the cytoskeleton actin (phalloidin, red) and the nuclei (TOPRO, blue) of hPDLSCs seeded on the CTRL and TEST group of samples taken after 48 h of culture established the cell adhesion. The cells adhered and spread well with a spindle fibroblast-like shape on both titanium surfaces, which confirmed that the different surface treatment did not affect the adhesion capability (Figure 4).

### Proliferation Assay of hPDLSCs Cultured on CTRL and TEST Titanium Surfaces

**FIGURE 5:**
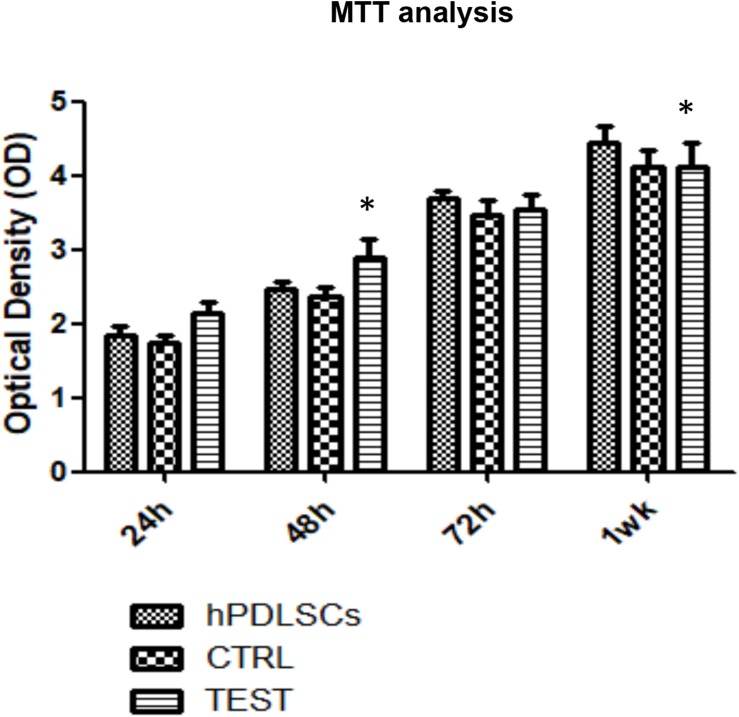
Proliferation assay of hPDLSCs seeded on CTRL and TEST titanium surfaces. MTT assay was evaluated at 24, 48, 72 h, and 1 week on both CTRL and TEST titanium surfaces. The human PDLSCs seeded on TEST surface showed after 24 and 48 h a slightly higher proliferation rate compared to cell cultured on CTRL surface and control cells. Cells cultured on CTRL and TEST surface after 72 h and 1 week of culture showed a similar proliferation rate of control cells. Proliferation was assessed using [3-(4,5-dimethyl-2-thiazolyl)-2,5-diphenyl-2H-tetrazoliumbromide test] (MTT) assay. The data shown are the mean (±SD) (*n* = 3). Densitometric values evaluated by ANOVA return significant differences, ^∗^*p* < 0.05.

MTT assay was performed at 24, 48, 72 h, and 1 week on both CTRL and TEST titanium surfaces. The hPDLSCs seeded on the TEST surface showed after 24 and 48 h a slightly higher proliferation rate compared to cells seeded on the CTRL surface and control cells. Cell cultured on the CTRL and TEST surface after 72 h and 1 week of culture showed a similar proliferation rate of control cells (Figure 5).

### Alizarin Red S Staining of hPDLSCs Grown in the Presence of CTRL and TEST Surfaces

**FIGURE 6:**
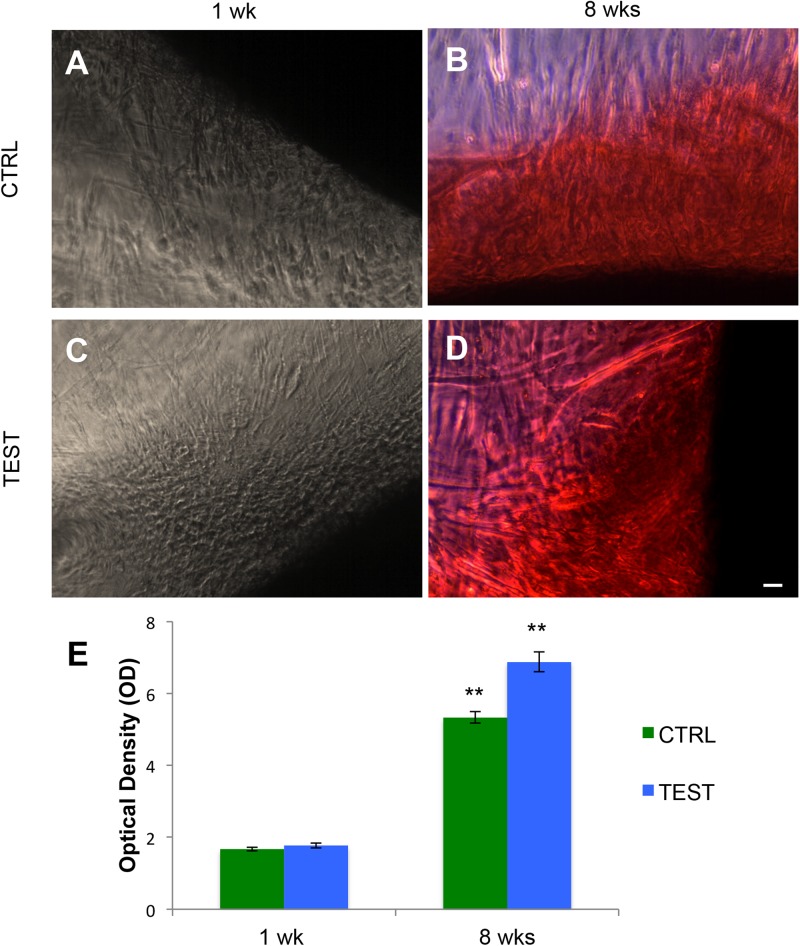
Human PDLSCs seeded on CTRL and TEST titanium implant surface were analyzed after 1 and 8 weeks of culture by Alizarin Red S staining (ARS). **(A)** Human PDLSCs seeded on CTRL and negative for Alizarin Red S (ARS) staining after 1 week of culture; **(B)** hPDLSCs cultured on CTRL and positive for ARS after 8 weeks of culture; **(C)** hPDLSCs seeded on TEST and negative for ARS; **(D)** hPDLSCs cultured on TEST and positive for ARS after 8 weeks of culture. ARS-positive staining was more evident in cells grown on TEST surface with respect to CTRL surface after 8 weeks of culture; **(E)** graph of ARS quantification. The error bars on these graphs showed standard deviation (±SD). Densitometric values analyzed by ANOVA return significant differences, **p < 0.01.

The osteogenic process in human PDLSCs, seeded with CTRL and TEST titanium surfaces after 1 week (Figures 6A,C) and after 8 weeks of culture (Figures 6B,D), was evaluated by Alizarin Red S (ARS) staining (Figures 6A–D) and its colorimetric detection (Figure 6E).

A significant augmented extracellular deposition was evident in cells grown after 8 weeks on the CTRL and TEST titanium surfaces compared to that of cells seeded on both surfaces after 1 week of culture (Figure 6).

### Titanium Surfaces Influence Protein Expression at CLSM

**FIGURE 7:**
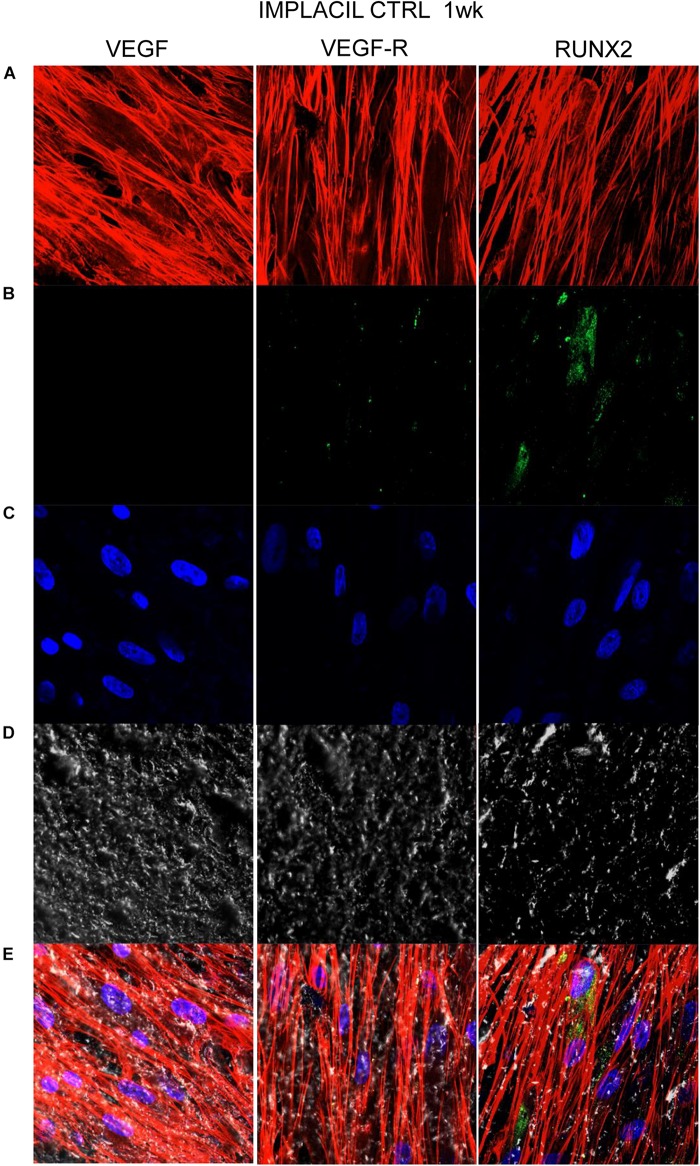
Human PDLSCs seeded on CTRL titanium implant surface were analyzed after 1 week of culture. **(A)** Cytoskeleton actin was stained in red fluorescence; **(B)** specific markers (VEGF, VEGF-R, and RUNX2) were stained in green fluorescence; **(C)** nuclei were stained in blue fluorescence. **(D)** PDLSCs with CTRL; **(E)** TL, transmission light: gray. Scale bar: 10 μm.

**FIGURE 8:**
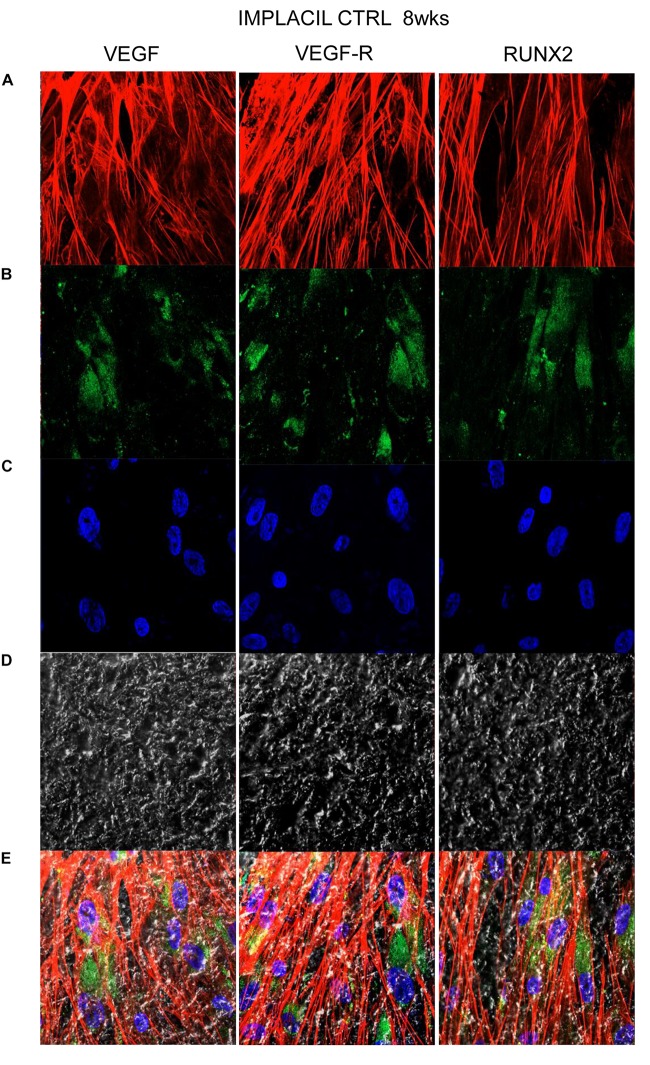
Human PDLSCs cultured on CTRL titanium implant surface were observed after 8 weeks of culture. **(A)** Cytoskeleton actin was stained in red fluorescence; **(B)** specific markers (VEGF, VEGF-R, and RUNX2) were stained in green fluorescence; **(C)** nuclei were stained in blue fluorescence. **(D)** PDLSCs with CTRL; **(E)** TL, transmission light: gray. Scale bar: 10 μm.

**FIGURE 9:**
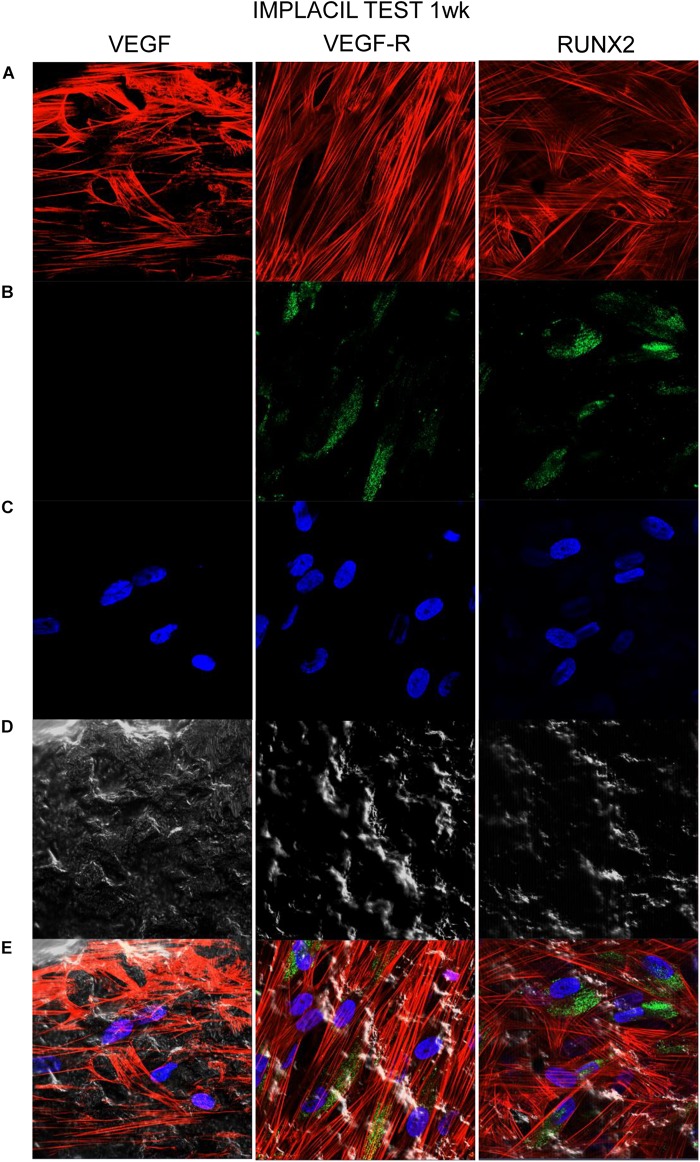
Human PDLSCs cultured on TEST titanium implant surface were observed after 1 week of incubation. **(A)** Cytoskeleton actin was stained in red fluorescence; **(B)** specific markers (VEGF, VEGF-R, and RUNX2) were stained in green fluorescence; **(C)** nuclei were stained in blue fluorescence. **(D)** PDLSCs with TEST; **(E)** TL, transmission light: gray. Scale bar: 10 μm.

**FIGURE 10:**
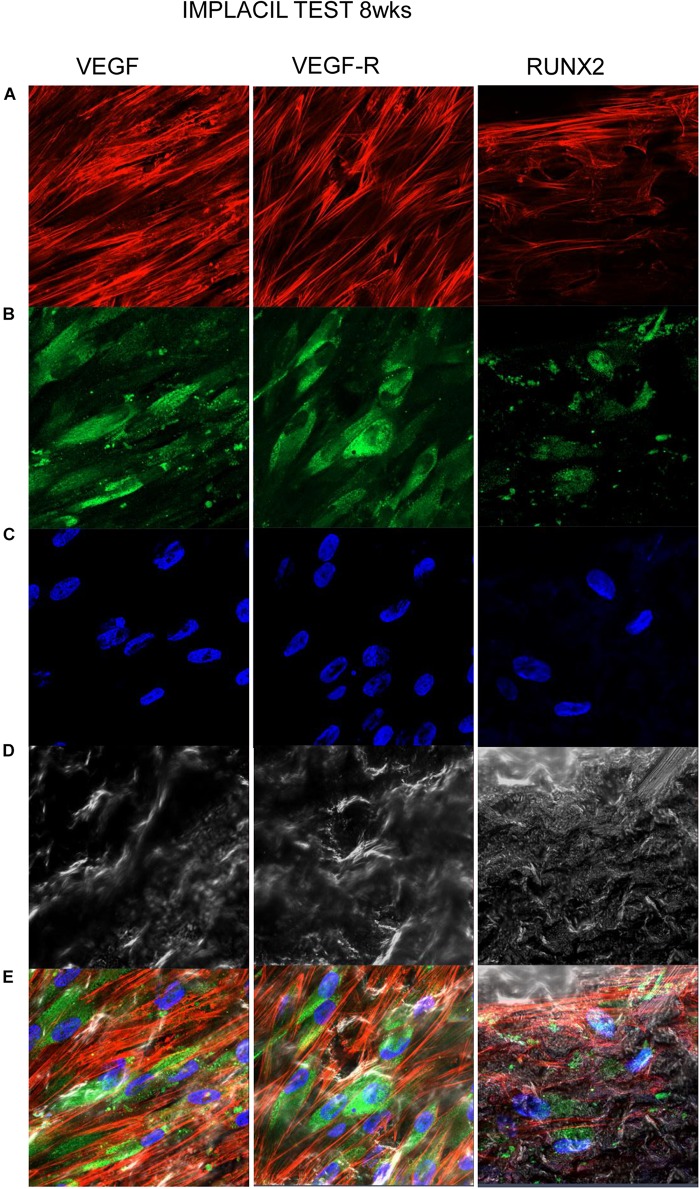
Human PDLSCs cultured on TEST titanium implant surface were observed after 8 weeks of incubation. **(A)** Cytoskeleton actin was stained in red fluorescence; **(B)** specific markers (VEGF, VEGF-R, and RUNX2) were stained in green fluorescence; **(C)** nuclei were stained in blue fluorescence. **(D)** PDLSCs with TEST; **(E)** TL, transmission light: gray. Scale bar: 10 μm.

**FIGURE 11:**
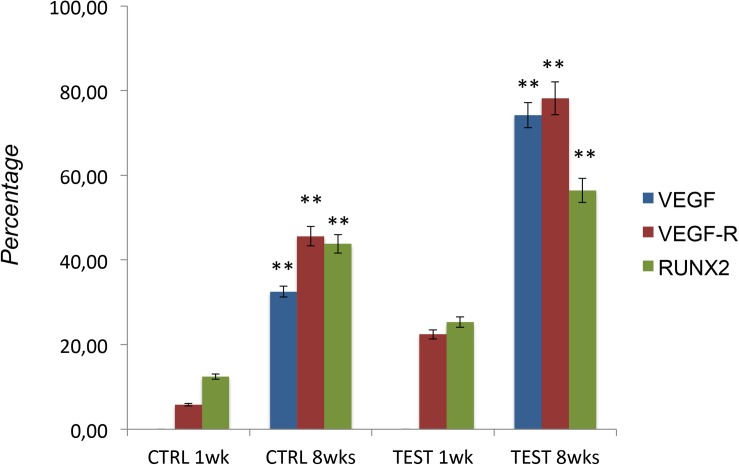
Graph bar evidence the percentage of positive cells for the specific markers (VEGF/VEGF-R/RUNX2). The error bars on these graphs showed standard deviation (±SD). ^∗∗^*p* < 0.01 significant difference of cells seeded on TEST and CTRL surfaces.

Figures 7–10 show fluorescence images of the cytoskeleton actin (phalloidin, red) and the nuclei (TOPRO, blue) of hPDLSCs cultured on the CTRL and TEST group of samples taken after 1 and 8 weeks of culture. The cells adhered and spread well with a spindle fibroblast-like shape on all samples, which indicates that the different surface treatment did not affect the cytocompatibility. The number of cells increased from 1 to 8 weeks (Figures 7–10). The CLSM observation demonstrated the tendency of osteogenic differentiation in cells cultured on TEST samples at 1 and 8 weeks of culture, which can be observed from the RUNX2-positive expression (Figures 9, 10). Cells seeded on TEST implant surface showed a positive expression for VEGF and VEGF-R when compared to cells cultured on CTRL samples (Figures 7–10). In particular, cells cultured on both surfaces after 8 weeks of culture evidenced a strong VEGF and VEGF-R expression compared to 1 week of culture. A qualitative analysis of fluorescent photomicrographs evidenced no expression of VEGF and a slow expression of VEGF-R and RUNX2 in cells seeded on CTRL and TEST samples after 1 week. Meanwhile, cells cultured on CTRL and TEST samples after 8 weeks of culture exhibited a high positivity of VEGF, VEGF-R, and RUNX2 (Figure 11).

### VEGF and RUNX2 Expression

**FIGURE 12:**
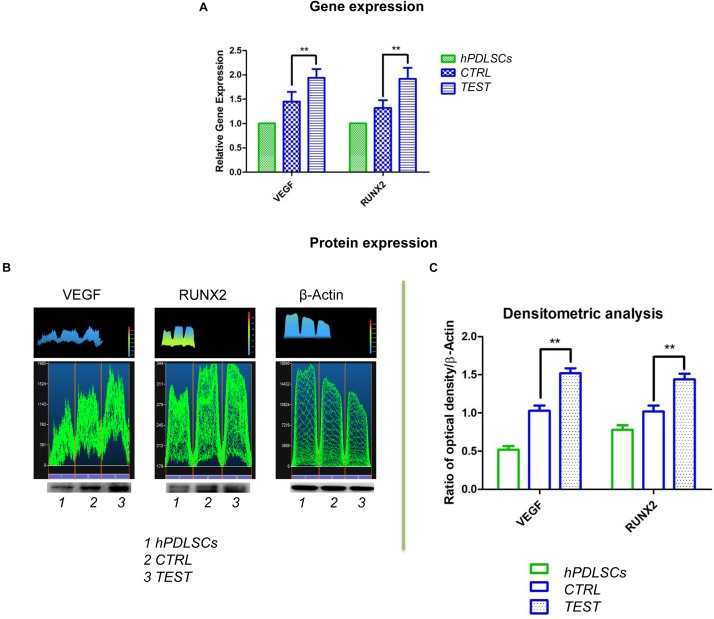
**(A)** Graph bar of RT-PCR showed the mRNA levels of VEGF and RUNX2 in cells seeded on CTRL and TEST surface after 8 weeks of culture ** *p* < 0.01. **(B)** Protein level expression of VEGF and RUNX2 in cells seeded on CTRL and TEST surface after 8 weeks of culture. **(C)** Graphs evidence densitometric analysis of protein bands expressed as integrated optical intensity (IOI) average of three separate experiments. The error bars on these graphs showed standard deviation (±SD). Densitometric values evaluated by ANOVA return significant differences, ** *p* < 0.01.

Histograms evidenced the gene expression of VEGF and RUNX2 performed by RT-PCR after 8 weeks of culture (Figure 12A). Human PDLSCs cultured on TEST exhibited an upregulation of VEGF and RUNX2 with respect to hPDLSCs seeded on the CTRL surface. Gene expression proved the qualitative data taken by CLSM analysis.

To identify protein expression of VEGF and RUNX2 in hPDLSCs seeded on CTRL and TEST after 8 weeks of culture, a Western blot analysis was performed. Protein expression of specific bands of VEGF and RUNX2 exhibited an over expression in hPDLSCs seeded on TEST with respect to the cells cultured on CTRL (Figure 12B and Supplementary Materials). Bar charts of densitometric analysis evidenced the related protein band quantification (Figure 12C). These results confirmed the obtained gene expression data.

## Discussion

Recently, novel treatment techniques of titanium surfaces were suggested and tested to increment the clinical performance, and to decrease the percentage of clinical unsuccessful attempts. The endeavors of scientific research were primarily concentrated to ameliorate the quantity and quality of bone tissue healing around implant surfaces and to increase the contact area between the implant surface and the bone tissue. All approaches were relatively surface treatments, adopted recently, driven to specific micro-structured surfaces with a better performance, liked to a greater contact area with the bone, have the capability to absorb biological molecules, induce more rapid healing, augmenting the cellular reaction and long-term clinical successes. Sandblasted and acid-etched surfaces have shown good biomechanical and biological results (Zizzari et al., 2015). Chemical modifications of titanium implant surfaces could be one active tool for achieving fast bone formation around the implant. After implant placement, several events occur between the host and the implant surface, which consist of the primary contact of blood with the implant surface, where proteins and ligands are vigorously adsorbed onto and released from the implant surface. As widely reported in the literature, the superficial oxide characteristics of the implant play an essential role in protein adsorption, and this fact is due to the biological tissues that cooperate primarily with the outside atomic layers of the dental implant. Surface chemistry, topography, roughness, and wettability of the surface could influence the type, quantity, and conformation of the incorporated protein layer. Different strategies including the modification of the surface physicochemical, morphological, and/or biochemical properties have been studied in order to increase the bone–implant interface (Shah et al., 2019). The usage of MSCs appears to promote encouraging outcomes in bone tissue renewal (Trubiani et al., 2019a). MSCs, taken from the oral cavity, showed differentiation ability and immunomodulatory characteristics (Fawzy El-Sayed and Dorfer, 2016). Based on the literature, hPDLSCs are encouraging dental stem cells and are utilized as a substitute to human bone marrow mesenchymal stem cells (BMMSCs) for evaluating the osteogenic capability of titanium surfaces and for assessing osseointegration in titanium implants (Zhou et al., 2016). For this motivation, the purpose of human PDLSCs cultured with the titanium implants represents a proper and appropriate exploratory approach to deeply study the mechanisms of cell interplay with distinct implantological surfaces (Gao et al., 2015).

In the current work, the observance was focused on the *in vitro* outcomes displayed by hPDLSCs cultured on diverse titanium implant surfaces (CTRL and TEST), established with two distinct methods, in order to assess the ability of the hPDLSCs to promote angiogenesis and osteogenesis. The present results have shown that hPDLSCs cultured on both experimental titanium disks presented a similar cell proliferation rate as the control cells (Figure 5). In particular, cells seeded on the TEST surface had a better performance, in terms of proliferation rate, when compared to the CTRL surfaces, after 48 h of culture (Figure 5). No significant differences between TEST and CTRL surfaces were observed under SEM, and the hPDLSCs revealed the same adhesion capability on both surfaces without any morphological changes (Figure 3). However, hPDLSCs seeded on TEST surfaces evidenced a greater osteogenic characteristic *in vitro* with respect to the cells seeded on CTRL surfaces. In detail, hPDLSCs in culture with TEST surface had an enhanced osteogenic response with an up-regulation of RUNX2, an early osteogenic marker. An upregulation of VEGF, VEGF-R, and RUNX2 was shown in both surfaces, after 8 weeks of culture, by Western blot (Figures 12B,C) and by CLSM analysis (Figures 8, 10). The protein expression of specific bands of VEGF and RUNX2 exhibited an over expression in hPDLSCs seeded on TEST with respect to the cells seeded on CTRL (Figures 12B,C). On the contrary, VEGF expression was not present, after 1 week of culture, on both surfaces under CLSM observations (Figures 7, 9). To further support these results, the gene expression of VEGF and RUNX2 was investigated through RT-PCR (Figure 12A). The data have shown that hPDLSCs cultured on TEST surfaces, after 8 weeks of culture, promoted an upregulation of VEGF and RUNX2 with respect to hPDLSCs cultured on CTRL surfaces (Figure 10). These results validated the data acquired by Western blot and under CLSM observations. Remarkably, a previous study evidenced that RUNX2 was a component of the genetic program that modulated the expression of VEGF throughout endochondral bone development (An et al., 2017). These data may encourage that the augmentation of RUNX2 could induce the enhanced VEGF and VEGF-R (Liu et al., 2012). The present data have shown that both titanium surfaces induced the increase of the pro-angiogenic factor VEGF, which plays an important and pivotal part in osteogenesis and bone regeneration, and particularly, the TEST surface showed a better performance with respect to the CTRL group. The relevant role of VEGF on osteogenesis has been previously established, and VEGF may regulate also calvaria ossification. Vascularization is a key process during osteogenesis and bone restoration (Lu et al., 2018). As largely reported in the literature, VEGF expression was evidenced during bone repair, indicating the key role of this angiogenic protein in bone tissue renewal. Furthermore, it was reported that VEGF administration in an experimental model of osteonecrosis of the femoral head led to bone remodeling and new bone formation (Dailiana et al., 2018; Jin et al., 2019). Blood vessels are an important component of bone formation and maintenance, and the rapid vascularization of tissue-engineered osteogenic grafts is a major obstacle in the development of regenerative medicine approaches for bone repair (Helmrich et al., 2013). The loss of VEGF in osterix-positive osteoblast progenitor cells caused a reduction in calvaria ossification, indicating that VEGF derived from these cells is required for optimal intramembranous bone formation (Duan et al., 2016). The reduction of VEGF injured endochondral bone formation, decreasing angiogenesis and osteogenesis, and causing a delay in fracture healing (Hu and Olsen, 2016).

The present results indicated that both surfaces, in contact with hPDLSCs, have shown not only osteoconductive properties, evaluated through the cell adhesion and proliferation, but also the ability to promote VEGF secretion from cells evidenced by gene expression and confocal microscopy. The induction of the release of VEGF and VEGF-R from hPDLSCs may represent an expectation for tissue engineering and, in particular, for the therapeutic growth of novel blood vessels surrounding the biomaterial in the first phase of osseointegration. In two recent studies, Raines et al. (2019a,b) have shown the production of pro-angiogenic substances by osteoblasts (Raines et al., 2019a, b), while Obice et al. (2019), in an *in vivo* human work, have established that the treatment with TiO_2_ of dental abutments increased the angiogenesis levels (Obice et al., 2019). In line with the augmentation of VEGF, VEGF-R, and RUNX2 by protein and gene expression, the confocal microscopy assessments exhibited the occurrence of molecules involved in vascularization events after 8 weeks of culture in hPDLSCs cultured on TEST. This TEST samples exhibited a better performance with regard to bone restorative ability. For the first time in the current work was investigated the VEGF and its receptor expression, a key player in angiogenesis, and RUNX2, early osteogenic marker on two novel implant titanium surfaces. The novelty of the current study is related to the properties evidenced of the titanium surfaces tested. It was shown that the surface topography of the TEST surface has a significant roughness with respect to the CTRL titanium surface. This property reported in our study is the primary fundamental and successful features on angiogenesis and osteogenesis due to augmenting the release of different pro-angiogenic growth components. Our study demonstrated that the TEST titanium surface promotes osteogenesis and angiogenesis, which has incredible prospective as a successful, engineered platform for bone tissue renewal. To conclude, these data have exhibited an essential role of the VEGF and its receptor, and a greater performance of TEST surfaces with respect to CTRL surfaces. The same data were described by Beltran-Partida et al. (2017) who found that rougher surfaces improved the endothelial proliferation (Beltran-Partida et al., 2017). This fact could be potentially useful in devising surfaces to be used in bone restoration processes and tissue engineering and, particularly, for the therapeutic development of novel blood vessels surrounding the material in the primary phase of osseointegration. The ability to augment the levels of VEGF and its receptor may conduct to faster bone–titanium integration.

## Data Availability Statement

The datasets generated for this study are available on request to the corresponding author.

## Ethics Statement

The studies involving human participants were reviewed and approved by the Medical Ethics Committee at the Medical School, “G. d’Annunzio” University, Chieti, Italy (no. 266/17.04.14). The patients/participants provided their written informed consent to participate in this study.

## Author Contributions

GM, FD, JP, and OT: conceptualization. LF and IM: formal analysis. FD, GM, and IM: investigation. GM, FD, LF, and IM: methodology. LF and JP: software. AP, OT, and SP: supervision. GM and FD: writing—original draft. EM, AP, SP, and OT: writing—review and editing.

## Conflict of Interest

The authors declare that the research was conducted in the absence of any commercial or financial relationships that could be construed as a potential conflict of interest.

## Supplementary Material

MATERIAL Western blot specific bands.

## References

[B1] An G. Zhang W. B. Ma D. K. Lu B. Wei G. J. Guang Y. (2017). Influence of VEGF/BMP-2 on the proliferation and osteogenetic differentiation of rat bone mesenchymal stem cells on PLGA/gelatin composite scaffold. *Eur. Rev. Med. Pharmacol. Sci.* 21 2316–2328. 28617560

[B2] Beltrán-Partida E. Valdéz-Salas B. Moreno-Ulloa A. Escamilla A. Curiel M. A. Rosales-Ibáñez R. (2017). Improved in vitro angiogenic behavior on anodized titanium dioxide nanotubes. *J. Nanobiotechnol.* 15:10 10.1186/s12951-017-0247-8 PMC5282661 28143540

[B3] Bianchi M. Pisciotta A. Bertoni L. Berni M. Gambardella A. Visani A. (2017). Corrigendum to “osteogenic differentiation of hDPSCs on biogenic bone apatite thin films”. *Stem Cells Int.* 2017:6587384 10.1155/2017/6587384 PMC5753003 29431156

[B4] Cavalcanti M. F. Maria D. A. de Isla N. Leal-Junior E. C. Joensen J. Bjordal J. M. (2015). Evaluation of the proliferative effects induced by low-level laser therapy in bone marrow stem cell culture. *Photomed. Laser Surg.* 33 610–616. 10.1089/pho.2014.3864 26580583

[B5] Conserva E. Pisciotta A. Borghi F. Nasi M. Pecorini S. Bertoni L. (2019). Titanium surface properties influence the biological activity and fasl expression of craniofacial stromal cells. *Stem Cells Int.* 2019:4670560 10.1155/2019/4670560 PMC6348805 30733806

[B6] Dailiana Z. H. Stefanou N. Khaldi L. Dimakopoulos G. Bowers J. R. Fink C. (2018). Vascular endothelial growth factor for the treatment of femoral head osteonecrosis: an experimental study in canines. *World J. Orthop.* 9 120–129. 10.5312/wjo.v9.i9.120 30254968 PMC6153136

[B7] De Colli M. Zara S. di Giacomo V. Patruno A. Marconi G. D. Gallorini M. (2015). Nitric oxide-mediated cytotoxic effect induced by zoledronic acid treatment on human gingival fibroblasts. *Clin. Oral Investig.* 19 1269–1277. 10.1007/s00784-014-1344-1349 25352469

[B8] Di Giulio C. Marconi G. D. Zara S. Di Tano A. Porzionato A. Pokorski M. (2015). Selective expression of galanin in neuronal-like cells of the human carotid body. *Adv. Exp. Med. Biol.* 860 315–323. 10.1007/978-3-319-18440-1_36 26303496

[B9] Di Nisio C. De Colli M. di Giacomo V. Rapino M. Di Valerio V. Marconi G. D. (2015). A dual role for beta1 integrin in an *in vitro* Streptococcus mitis/human gingival fibroblasts co-culture model in response to TEGDMA. *Int. Endod J.* 48 839–849. 10.1111/iej.12379 25231818

[B10] Diomede F. D’Aurora M. Gugliandolo A. Merciaro I. Ettorre V. Bramanti A. (2018a). A novel role in skeletal segment regeneration of extracellular vesicles released from periodontal-ligament stem cells. *Int. J. Nanomed.* 13 3805–3825. 10.2147/Ijn.S162836 PMC6029600 29988728

[B11] Diomede F. D’Aurora M. Gugliandolo A. Merciaro I. Orsini T. Gatta V. (2018b). Biofunctionalized scaffold in bone tissue repair. *Int. J. Mol. Sci.* 19:1022 10.3390/ijms19041022 PMC5979468 29596323

[B12] Diomede F. Gugliandolo A. Scionti D. Merciaro I. Cavalcanti M. F. Mazzon E. (2018c). Biotherapeutic effect of gingival stem cells conditioned medium in bone tissue restoration. *Int. J. Mol. Sci.* 19:329 10.3390/ijms19020329 PMC5855551 29360771

[B13] Diomede F. Merciaro I. Martinotti S. Cavalcanti M. F. Caputi S. Mazzon E. (2016). miR-2861 is involved in osteogenic commitment of human periodontal ligament stem cells grown onto 3D scaffold. *J. Biol. Regul. Homeost. Agents* 30 1009–1018. 28078846

[B14] Diomede F. Zini N. Pizzicannella J. Merciaro I. Pizzicannella G. D’Orazio M. (2018d). 5-Aza exposure improves reprogramming process through embryoid body formation in human gingival stem cells. *Front. Genet.* 9:419 10.3389/fgene.2018.00419 PMC6186780 30349553

[B15] Duan X. Bradbury S. R. Olsen B. R. Berendsen A. D. (2016). VEGF stimulates intramembranous bone formation during craniofacial skeletal development. *Matrix Biol.* 52-54 127–140. 10.1016/j.matbio.2016.02.005 26899202 PMC4875795

[B16] Fawzy El-Sayed K. M. Dorfer C. E. (2016). Gingival mesenchymal stem/progenitor cells: a unique tissue engineering gem. *Stem Cells Int.* 2016:7154327 10.1155/2016/7154327 PMC4903147 27313628

[B17] Gao H. Li B. Zhao L. Jin Y. (2015). Influence of nanotopography on periodontal ligament stem cell functions and cell sheet based periodontal regeneration. *Int. J. Nanomedicine* 10 4009–4027. 10.2147/IJN.S83357 26150714 PMC4484652

[B18] Genova T. Petrillo S. Zicola E. Roato I. Ferracini R. Tolosano E. (2019). The crosstalk between osteodifferentiating stem cells and endothelial cells promotes angiogenesis and bone formation. *Front. Physiol.* 10:1291 10.3389/fphys.2019.01291 PMC6802576 31681005

[B19] Giacoppo S. Thangavelu S. R. Diomede F. Bramanti P. Conti P. Trubiani O. (2017). Anti-inflammatory effects of hypoxia-preconditioned human periodontal ligament cell secretome in an experimental model of multiple sclerosis: a key role of IL-37. *FASEB J.* 31 5592–5608. 10.1096/fj.201700524R 28842429 PMC5690382

[B20] Gittens R. A. Scheideler L. Rupp F. Hyzy S. L. Geis-Gerstorfer J. Schwartz Z. (2014). A review on the wettability of dental implant surfaces II: biological and clinical aspects. *Acta Biomater.* 10 2907–2918. 10.1016/j.actbio.2014.03.032 24709541 PMC4103435

[B21] Gugliandolo A. Diomede F. Cardelli P. Bramanti A. Scionti D. Bramanti P. (2018). Transcriptomic analysis of gingival mesenchymal stem cells cultured on 3D bioprinted scaffold: a promising strategy for neuroregeneration. *J. Biomed. Mater. Res. A* 106 126–137. 10.1002/jbm.a.36213 28879677

[B22] Hanawa T. (2019). Titanium-tissue interface reaction and its control with surface treatment. *Front. Bioeng. Biotechnol.* 7:170 10.3389/fbioe.2019.00170 PMC6650641 31380361

[B23] Helmrich U. Di Maggio N. Guven S. Groppa E. Melly L. Largo R. D. (2013). Osteogenic graft vascularization and bone resorption by VEGF-expressing human mesenchymal progenitors. *Biomaterials* 34 5025–5035. 10.1016/j.biomaterials.2013.03.040 23566801

[B24] Hu K. Olsen B. R. (2016). The roles of vascular endothelial growth factor in bone repair and regeneration. *Bone* 91 30–38. 10.1016/j.bone.2016.06.013 27353702 PMC4996701

[B25] Jin X. Han D. Tao J. Huang Y. Zhou Z. Zhang Z. (2019). Dimethyloxallyl glycine-incorporated borosilicate bioactive glass scaffolds for improving angiogenesis and osteogenesis in critical-sized calvarial defects. *Curr. Drug Deliv.* 16 565–576. 10.2174/1567201816666190611105205 31198114

[B26] Kado T. Aita H. Ichioka Y. Endo K. Furuichi Y. (2019). Chemical modification of pure titanium surfaces to enhance the cytocompatibility and differentiation of human mesenchymal stem cells. *Dent. Mater. J.* 38 1026–1035. 10.4012/dmj.2018-2257 31582594

[B27] Kim S. Y. Yoo J. Y. Ohe J. Y. Lee J. W. Moon J. H. Kwon Y. D. (2014). Differential expression of osteo-modulatory molecules in periodontal ligament stem cells in response to modified titanium surfaces. *Biomed. Res. Int.* 2014:452175 10.1155/2014/452175 PMC4095730 25057487

[B28] Libro R. Scionti D. Diomede F. Marchisio M. Grassi G. Pollastro F. (2016). Cannabidiol modulates the immunophenotype and inhibits the activation of the inflammasome in human gingival mesenchymal stem cells. *Front. Physiol.* 7:559 10.3389/fphys.2016.00559 PMC5121123 27932991

[B29] Liu X. Chen S. Tsoi J. K. H. Matinlinna J. P. (2017). Binary titanium alloys as dental implant materials-a review. *Regen. Biomater.* 4 315–323. 10.1093/rb/rbx027 29026646 PMC5633690

[B30] Liu Y. Berendsen A. D. Jia S. Lotinun S. Baron R. Ferrara N. (2012). Intracellular VEGF regulates the balance between osteoblast and adipocyte differentiation. *J. Clin. Invest.* 122 3101–3113. 10.1172/JCI61209 22886301 PMC3428080

[B31] Lu L. Deegan A. Musa F. Xu T. Yang Y. (2018). The effects of biomimetically conjugated VEGF on osteogenesis and angiogenesis of MSCs (human and rat) and HUVECs co-culture models. *Colloids Surf. B Biointerfaces* 167 550–559. 10.1016/j.colsurfb.2018.04.060 29730577

[B32] Mammana S. Gugliandolo A. Cavalli E. Diomede F. Iori R. Zappacosta R. (2019). Human gingival mesenchymal stem cells pretreated with vesicular moringin nanostructures as a new therapeutic approach in a mouse model of spinal cord injury. *J. Tissue Eng. Regen. Med.* 13 1109–1121. 10.1002/term.2857 30942960 PMC6771565

[B33] Mastropasqua L. Toto L. D’Ugo E. Lanzini M. Mattei P. A. Falconio G. (2020). In vivo and in vitro results of an automated preloaded delivery system for IOL implantation in cataract surgery. *Int. Ophthalmol.* 40 125–134. 10.1007/s10792-019-01154-0 31451986

[B34] Mazzatenta A. Marconi G. D. Macchi V. Porzionato A. Cataldi A. Di Giulio C. (2016). Coexpression of galanin and nestin in the chemoreceptor cells of the human carotid body. *Adv. Exp. Med. Biol.* 885 77–82. 10.1007/5584_2015_189 26747071

[B35] Mazzatenta A. Marconi G. D. Zara S. Cataldi A. Porzionato A. Di Giulio C. (2014). In the carotid body, galanin is a signal for neurogenesis in young, and for neurodegeneration in the old and in drug-addicted subjects. *Front. Physiol.* 5:427 10.3389/fphys.2014.00427 PMC4215693 25400591

[B36] Obice A. L. S. Correa M. G. Feng H. S. Ribeiro F. V. Cirano F. R. Casati M. Z. (2019). The impact of implant abutment surface treatment with TiO2 on peri-implant levels of angiogenesis and bone-related markers: a randomized clinical trial. *Int. J. Oral Maxillofac. Surg.* 48 962–970. 10.1016/j.ijom.2018.12.012 30661944

[B37] Pizzicannella J. Cavalcanti M. Trubiani O. Diomede F. (2018a). MicroRNA 210 mediates VEGF upregulation in human periodontal ligament stem cells cultured on 3DHydroxyapatite ceramic scaffold. *Int. J. Mol. Sci.* 19:3916 10.3390/ijms19123916 PMC6320762 30563289

[B38] Pizzicannella J. Diomede F. Gugliandolo A. Chiricosta L. Bramanti P. Merciaro I. (2019a). 3D Printing PLA/Gingival Stem Cells/EVs Upregulate miR-2861 and -210 during osteoangiogenesis commitment. *Int. J. Mol. Sci.* 20:3256 10.3390/ijms20133256 PMC6651609 31269731

[B39] Pizzicannella J. Diomede F. Merciaro I. Caputi S. Tartaro A. Guarnieri S. (2018b). Endothelial committed oral stem cells as modelling in the relationship between periodontal and cardiovascular disease. *J. Cell Physiol.* 233 6734–6747. 10.1002/jcp.26515 29600566

[B40] Pizzicannella J. Gugliandolo A. Orsini T. Fontana A. Ventrella A. Mazzon E. (2019b). Engineered extracellular vesicles from human periodontal-ligament stem cells increase VEGF/VEGFR2 expression during bone regeneration. *Front. Physiol.* 10:512 10.3389/fphys.2019.00512 PMC6503111 31114512

[B41] Raines A. L. Berger M. B. Patel N. Hyzy S. L. Boyan B. D. Schwartz Z. (2019a). VEGF-A regulates angiogenesis during osseointegration of Ti implants via paracrine/autocrine regulation of osteoblast response to hierarchical microstructure of the surface. *J. Biomed. Mater. Res. A* 107 423–433. 10.1002/jbm.a.36559 30461195 PMC6892345

[B42] Raines A. L. Berger M. B. Schwartz Z. Boyan B. D. (2019b). Osteoblasts grown on microroughened titanium surfaces regulate angiogenic growth factor production through specific integrin receptors. *Acta Biomater.* 97 578–586. 10.1016/j.actbio.2019.07.036 31349056 PMC7250132

[B43] Rajan T. S. Giacoppo S. Trubiani O. Diomede F. Piattelli A. Bramanti P. (2016). Conditioned medium of periodontal ligament mesenchymal stem cells exert anti-inflammatory effects in lipopolysaccharide-activated mouse motoneurons. *Exp. Cell Res.* 349 152–161. 10.1016/j.yexcr.2016.10.008 27737733

[B44] Romeo L. Diomede F. Gugliandolo A. Scionti D. Lo Giudice F. Cariccio V. L. (2018). Moringin induces neural differentiation in the stem cell of the human periodontal ligament. *Sci. Rep.* 8:9153 10.1038/S41598-018-27492-27490 PMC6002387 29904155

[B45] Safi I. N. Mohammed Ali Hussein B. Al-Shammari A. M. (2019). In vitro periodontal ligament cell expansion by co-culture method and formation of multi-layered periodontal ligament-derived cell sheets. *Regen. Ther.* 11 225–239. 10.1016/j.reth.2019.08.002 31528667 PMC6739433

[B46] Selders G. S. Fetz A. E. Radic M. Z. Bowlin G. L. (2017). An overview of the role of neutrophils in innate immunity, inflammation and host-biomaterial integration. *Regen. Biomater.* 4 55–68. 10.1093/rb/rbw041 28149530 PMC5274707

[B47] Shah F. A. Thomsen P. Palmquist A. (2019). Osseointegration and current interpretations of the bone-implant interface. *Acta Biomater.* 84 1–15. 10.1016/j.actbio.2018.11.018 30445157

[B48] Sinjari B. Pizzicannella J. D’Aurora M. Zappacosta R. Gatta V. Fontana A. (2019). Curcumin/liposome nanotechnology as delivery platform for anti-inflammatory activities via NFkB/ERK/pERK pathway in human dental pulp treated With 2-hydroxyethyl methacrylate (HEMA). *Front. Physiol.* 10:633 10.3389/fphys.2019.00633 PMC6579913 31244665

[B49] Trubiani O. Ballerini P. Murmura G. Pizzicannella J. Giuliani P. Buccella S. (2012a). Toll-like receptor 4 expression, interleukin-6, -8 and Ccl-20 release, and NF-KB translocation in human periodontal ligament mesenchymal stem cells stimulated with LPS-P. *Gingivalis*. *Eur. J. Inflammation* 10 81–89. 10.1177/1721727x1201000109

[B50] Trubiani O. Marconi G. D. Pierdomenico S. D. Piattelli A. Diomede F. Pizzicannella J. (2019a). Human oral stem cells, biomaterials and extracellular vesicles: a promising tool in bone tissue repair. *Int. J. Mol. Sci.* 20:4987 10.3390/ijms20204987 PMC6834314 31600975

[B51] Trubiani O. Pizzicannella J. Caputi S. Marchisio M. Mazzon E. Paganelli R. (2019b). Periodontal ligament stem cells: current knowledge and future perspectives. *Stem Cells Dev.* 28 995–1003. 10.1089/scd.2019.0025 31017047

[B52] Trubiani O. Toniato E. Di Iorio D. Diomede F. Merciaro I. (2012b). Morphological analysis and interleukin release in human gingival fibroblasts seeded on different denture base acrylic resins. *Int. J. Immunopathol. Pharmacol.* 25 637–643. 10.1177/039463201202500310 23058014

[B53] Winning L. Robinson L. Boyd A. R. El Karim I. A. Lundy F. T. Meenan B. J. (2017). Osteoblastic differentiation of periodontal ligament stem cells on non-stoichiometric calcium phosphate and titanium surfaces. *J. Biomed. Mater. Res. A* 105 1692–1702. 10.1002/jbm.a.36044 28218482

[B54] Yadav A. Yadav R. Gupta A. Baranwal A. Bhatnagar A. Singh V. (2017). Effect of ultraviolet irradiation on the osseointegration of a titanium alloy with bone. *Contemp. Clin. Dent.* 8 571–578. 10.4103/ccd.ccd_576_17 29326508 PMC5754978

[B55] Zahran R. Rosales Leal J. I. Rodriguez Valverde M. A. Cabrerizo Vilchez M. A. (2016). Effect of hydrofluoric acid etching time on titanium topography, chemistry, wettability, and cell adhesion. *PLoS One* 11:e0165296 10.1371/journal.pone.0165296 PMC5100918 27824875

[B56] Zara S. De Colli M. Rapino M. Di Valerio V. Marconi G. D. Cataldi A. (2013). NF-kappaB involvement in hyperoxia-induced myocardial damage in newborn rat hearts. *Histochem. Cell Biol.* 140 575–583. 10.1007/s00418-013-1092-y 23568329

[B57] Zhou Q. Yang P. Li X. Liu H. Ge S. (2016). Bioactivity of periodontal ligament stem cells on sodium titanate coated with graphene oxide. *Sci. Rep.* 6:19343 10.1038/srep19343 PMC4725920 26763307

[B58] Zizzari V. L. Marconi G. D. De Colli M. Zara S. Zavan B. Salini V. (2015). *In vitro* behavior of primary human osteoblasts onto microrough titanium surface. *Implant Dent.* 24 377–383. 10.1097/ID.0000000000000268 25915409

