# Supplementary material for: Enhanced VEGF/VEGF-R and RUNX2 Expression in Human Periodontal Ligament Stem Cells Cultured on Sandblasted/Etched Titanium Disk
Source: Front Cell Dev Biol. 2020 May 14;8:315. doi: 10.3389/fcell.2020.00315 (PMC7240029; doi:10.3389/fcell.2020.00315)
Supplement: MATERIAL — Western blot specific bands. [file Data_Sheet_1.PDF]

β-actin

1 2 3

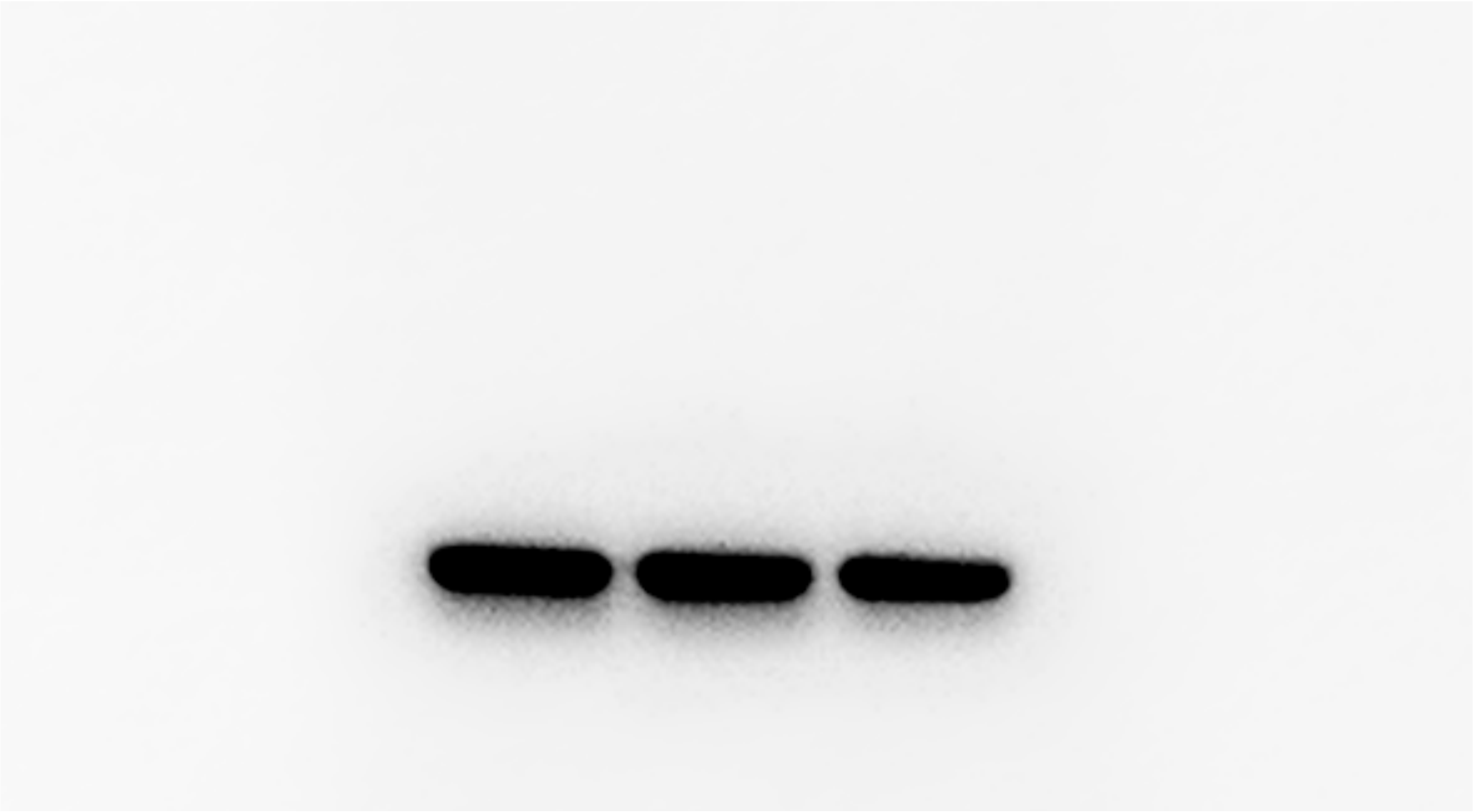

1 hPDLSCs
2 CTRL
3 TEST

8 % gel polyacrylamide

VEGF

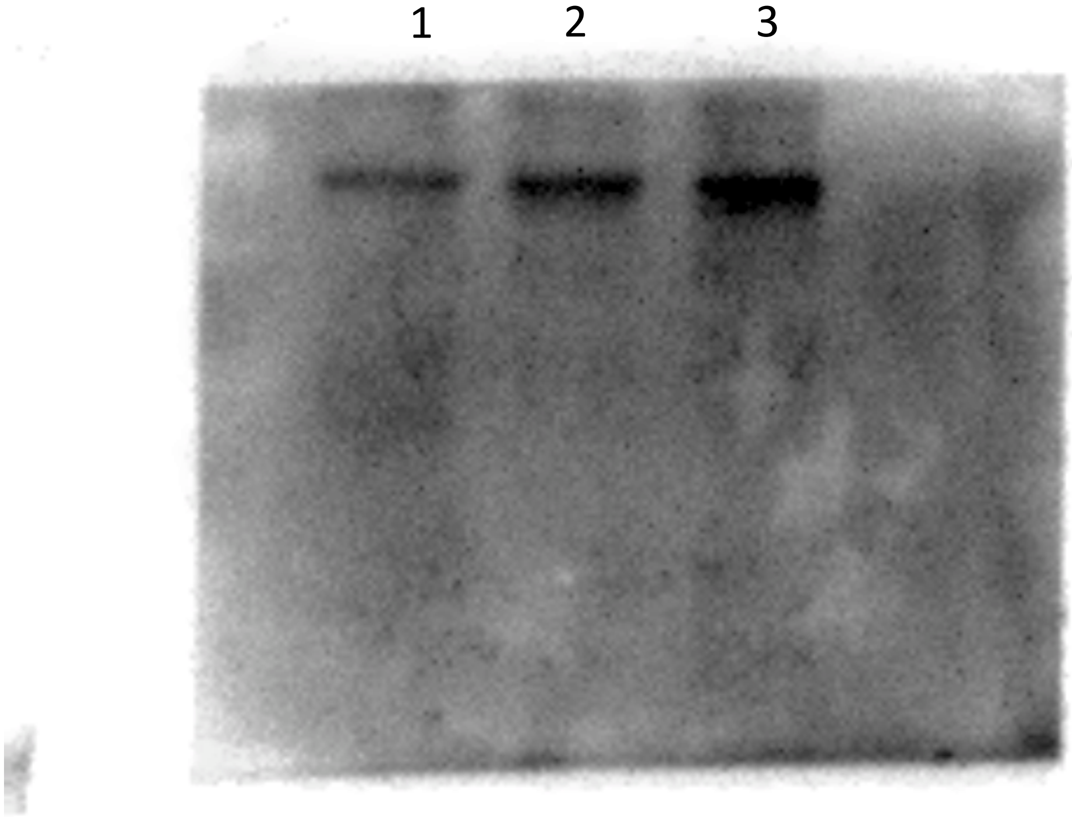

1 hPDLSCs
2 CTRL
3 TEST

12 % gel polyacrylamide

RUNX2

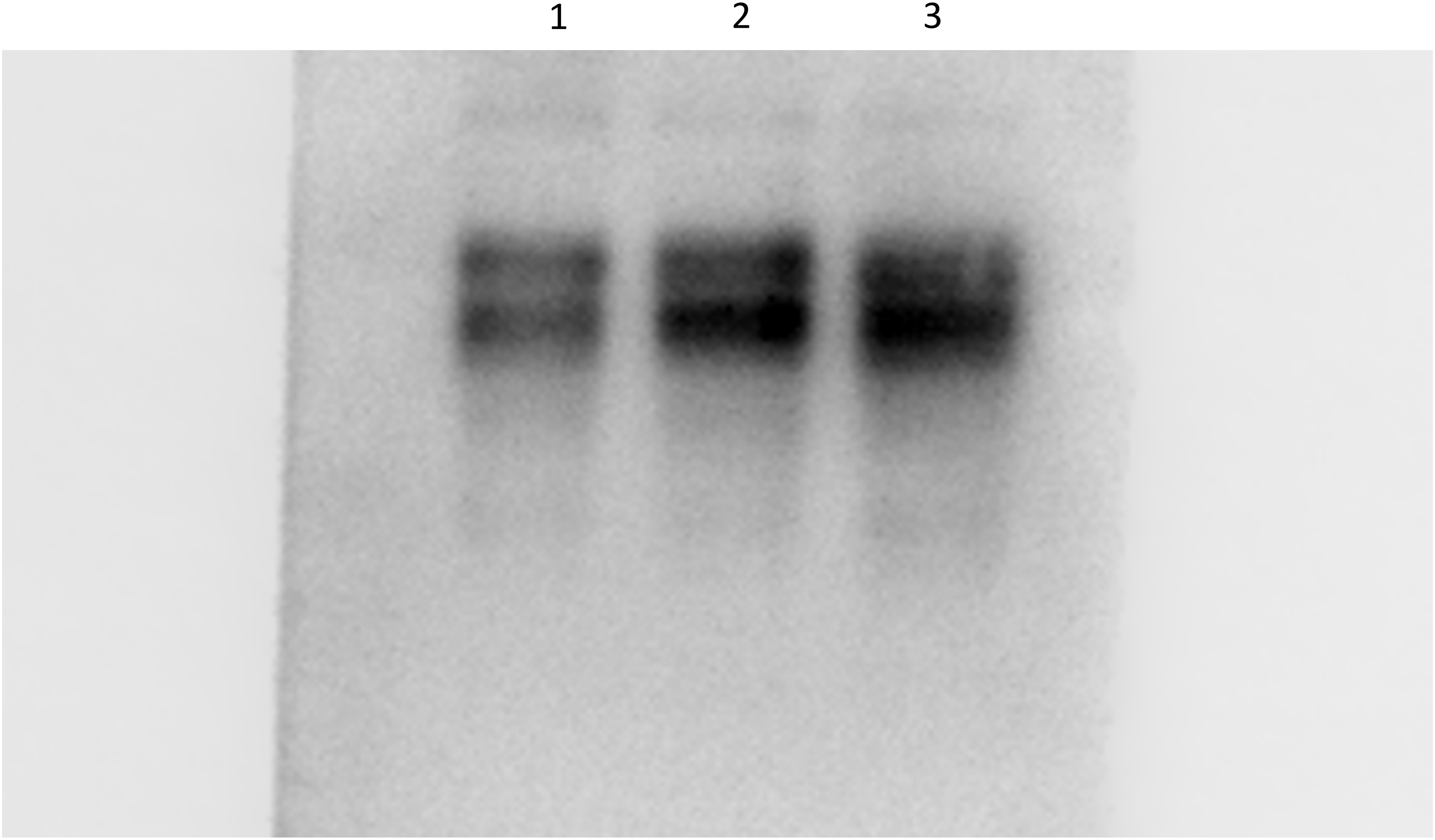

1 hPDLSCs
2 CTRL
3 TEST

8 % gel polyacrylamide
